# Enhancement of Bioactive Compounds in “Valencia” Orange Peel Waste During Cold Storage by Melatonin and Arginine Treatments

**DOI:** 10.1002/fsn3.70448

**Published:** 2025-06-27

**Authors:** Faezeh Aghaei, Asghar Ramezanian, Mohammad Jamal Saharkhiz, Mohammad‐Taghi Golmakani, Vahid Rowshan

**Affiliations:** ^1^ Department of Horticultural Science, School of Agriculture Shiraz University Shiraz Iran; ^2^ Department of Food Science and Technology, School of Agriculture Shiraz University Shiraz Iran; ^3^ Department of Natural Resources Fars Agricultural and Natural Resources Research and Education Center, AREEO Shiraz Iran

**Keywords:** arginine, bioactive compounds, citrus, melatonin

## Abstract

This study was carried out to investigate the protective effects of melatonin (Mel) and arginine (Arg) treatments on the maintenance and enhancement of bioactive compounds in Valencia orange peel, including soluble sugars, organic acids, and phenolic compounds, during 6 months of cold storage. This research was conducted to optimize storage conditions and increase the added value of orange peel as a rich source of functional compounds in the food and pharmaceutical industries. The 1 mM‐Mel treatment increased fructose by 14%. Glucose content decreased in the control, 2 mM‐Mel, 5 mM‐Arg, and 10 mM‐Arg treatments, but remained constant at 1 mM‐Mel. Sucrose content increased by 46% at 10 mM‐Arg. Ascorbic acid increased by 145% with 1 mM‐Mel and by 90% with 10 mM‐Arg. Citric acid increased by 173% with 1 mM‐Mel and by 102% with 1 mM‐Arg. Coumaric acid increased by 44% with 1 mM‐Mel and remained constant at 10 mM‐Arg. *Trans*‐ferulic acid increased by 50% with 5 mM‐Arg. Chlorogenic acid increased by 103% with 1 mM‐Mel. Catechin increased by 15% with 10 mM‐Arg. Quercetin increased by 292% with 10 mM‐Arg. Hesperidin increased by 35% with 10 mM‐Arg and hesperetin by 74% with 10 mM‐Arg. Mel and Arg maintained the bioactive compounds of orange peel during cold storage. They are potential treatments to increase the added value of agricultural products by preserving bioactive compounds.

## Graphical abstract

This study evaluated melatonin and arginine effects on bioactive compounds in Valencia orange peel during 6 months of cold storage. Mel (1 mM) enhanced fructose, ascorbic, citric, and chlorogenic acids, whereas Arg (10 mM) increased sucrose, ascorbic acid, and phenolic compounds. Both treatments preserved and enhanced bioactive compounds in orange peel.

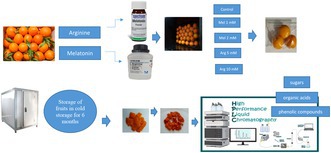

## 1 Introduction

Citrus fruits, belonging to the Rutaceae family, are mainly grown in tropical and subtropical regions regions due to favorable soil and climate conditions. Flavonoids (hesperidin, hesperetin, naringin, naringenin, coumarins, quercetin), phenolic acids, terpenoids (carotenoids and limonoids), dietary fibers (like pectin), minerals (iron, manganese, zinc, selenium, and copper), and vitamins (A, B, C, and E, including thiamine, riboflavin, and niacin) are all abundant in them (Riaz et al. 2023). The risk of diabetes, cancer, and cardiovascular diseases is considerably decreased by these bioactive substances (Ahmed and Saeid 2021).

Oranges ( *Citrus sinensis* ) account for almost half of the global citrus production, making them the most produced and consumed citrus fruit worldwide (Seminara et al. 2023). Among the different types of oranges, the Valencia variety is primarily used for juice, jam, and pulp production due to its adaptability to different climatic conditions (Ortiz‐Sanchez et al. 2024). Typically, during orange juice production, about 50% of the fruit's weight comprises orange juice, whereas the remaining 50% consists of pulp, peels, and seeds, often regarded as waste (Dassoff et al. 2021). Orange peels account for 60%–65% of the 8–20 million tons of orange waste produced annually from orange juice production (Mohsin et al. 2022). Consequently, orange peels represent the most portion and manageable waste by volume in the orange industry, leading to significant environmental challenges. The disposal of orange peel waste in landfills results in significant greenhouse gas emissions and produces leachates with high chemical oxygen demand (Ortiz‐Sanchez et al. 2024). On the other hand, recent study highlights the environmental challenges posed by the high moisture content of orange peels. It discusses how orange peels, with their significant moisture levels, can serve as a substrate for microbes, flies, mold, and mycotoxins growth, leading to environmental hazards. The study also explores the potential of orange peels in biotechnological applications, such as solid‐state fermentation, to enhance their nutritional value and reduce waste (Araújo et al. 2024). The rapid degradation and large volume of orange peel waste create significant waste management challenges, leading to environmental concerns and lost resources. This waste represents a missed opportunity, as it contains valuable bioactive compounds applicable to various industries, thereby incurring both ecological and economic disadvantages (Junker et al. 2024). Numerous factors affect the chemical composition of orange peel waste, such as variety, ripening, postharvest conditions, soil properties, and methods used to produce orange juice (Ortiz‐Sanchez et al. 2024). Therefore, further research is needed to preserve the quality of orange peel waste and enhance the production of high‐value‐added products before utilizing these renewable resources in various fields.

The use of environmentally friendly natural substances or biostimulants has recently emerged as a promising approach for maintaining the quality of postharvest horticultural crops (Nasr et al. 2022). Recent studies highlight the potential of melatonin in postharvest treatments for citrus peels. For example, melatonin application at 0.1 mmol/L significantly increased the accumulation of polymethoxylated flavones in citrus fruits, enhancing their bioactive properties and regulating flavonoid biosynthesis through gene expression changes. This suggests its role in improving the nutritional and pharmaceutical value of citrus peels during storage (Zhao et al. 2024). Numerous investigations have demonstrated that treating fruits such as mangoes, pineapples, and bananas with melatonin at concentrations of 1 and 2 mM leads to a reduction in decay, preservation of quality, and extension of their longevity under both cold storage and ambient conditions (Anchana et al. 2023; Charoenphun et al. 2025; Mandal et al. 2024). For instance, Zhang et al. (2018) revealed that melatonin, by diminishing the activity of polyphenol oxidase (PPO) enzyme, effectively prevents the browning of lychee fruit. Furthermore, Magri and Petriccione (2022) reported that a 1 mM‐Mel treatment of blueberries curtailed the activity of oxidative enzymes while augmenting antioxidant compounds. Marak et al. (2024) also corroborated that melatonin delays decay and browning in lychee fruit by attenuating PPO enzyme activity. The selection of these concentrations for our study was predicated upon the positive outcomes observed in previous research. Furthermore, arginine has been the subject of numerous scientific investigations as an effective compound for preserving quality and extending the postharvest shelf life of fruits. Li, Xu, et al. (2019) reported that arginine, particularly at elevated concentrations of 6 and 12 mM, significantly inhibited the growth of Botrytis cinerea in cherry tomatoes, whereas mitigating oxidative damage through the enhancement of the antioxidant system. Wang et al. (2023) also demonstrated that arginine maintained the quality of blueberries by reducing oxidative damage and promoting antioxidant system activity, thereby improving their weight, firmness, and soluble solid content. However, it is noteworthy that Shi et al. (2022) documented the beneficial effects of arginine, even at a lower concentration of 1 mM, on the quality and shelf life of soft‐seeded and hard‐seeded pomegranate arils during cold storage, preventing the reduction of vitamin C, phenolic compounds, and anthocyanins. Given the paucity of prior studies on citrus fruits, this research was conducted to determine the optimal concentration range of arginine by examining the effects of 5 and 10 mM concentrations, selected based on the positive effects reported in other fruits, on the peel quality of citrus fruits. Research has shown that applications of melatonin and arginine can preserve the nutritional value and extend the storage life of horticultural crops (Nasr et al. 2022). To the best of our knowledge, there are no reports on the impact of postharvest application of these compounds on the quality of citrus peel. Therefore, to better understand how varying melatonin and arginine concentrations affect the bioactive compounds in the “Valencia” orange peel during cold storage, this study was conducted.

## 2 Materials and Methods

### 2.1 Plant Material

“Valencia” orange fruits, harvested at commercial maturity based on TSS/TA ratio from a commercial orchard in Darab, Fars, Iran (latitude 28°45′ N, longitude 54°33′ E, altitude 1170 m), were immediately postharvest meticulously sorted and graded in specialized baskets to prevent excessive pressure. Subsequently, within 3 h, the fruit were transported to the laboratory at Shiraz University under an ambient temperature of 23°C using a passenger vehicle, where they underwent experimental treatments and further postharvest analyses.

### 2.2 Postharvest Treatment and Experimental Design

Uniform, damage‐ and decay‐free fruit were meticulously selected. Subsequently, surface sterilization was performed by immersing the fruit in a 2% v/v sodium hypochlorite solution for 20 min, followed by thorough rinsing with distilled water and air‐drying at 20°C for 20 min. The experiment was conducted using a completely randomized design with three replicates. Fruit were treated by immersing in either melatonin (1 and 2 mM) and arginine (5 and 10 mM) solutions for 20 min. Control fruit were immersed in distilled water. Throughout the treatment process, a consistent laboratory temperature of 20°C was maintained. Additionally, treatment solutions of arginine and melatonin were prepared and stored at 20°C to ensure uniform and controlled experimental conditions.

### 2.3 Storage Condition

The nylon bags used for storage were hermetically sealed. Subsequently, the sealed bags were stored in a controlled environment at 5°C with a relative humidity maintained between 85% and 95% for 6 months. Under these conditions, the relative humidity within the bags was also maintained within the same range. At the beginning and end of the storage period, undamaged fruits were washed with distilled water and peeled using a small knife. The fresh orange peel (flavo and albedo) was cut into 1 cm^2^ squares and dried in a cool, shaded place away from sunlight and any contamination, with air flowing between the samples, for 72 h at 25°C. The samples were stored at 4°C until use.

### 2.4 Extraction of Soluble Sugars, Organic Acids, and Phenolic Compounds

The method of De Miera et al. (2023) was used with slight modifications to extract soluble sugars, organic acids, and phenolic compounds. A ball mill was used to pulverize the dried orange peels. Subsequently, 0.7 g of the powdered samples were mixed with 7 mL of HPLC‐grade methanol, resulting in a biomass‐to‐solvent ratio of 1:10 (g/mL). Extractions were performed at 25°C for 45 min using a 300 W ultrasonic homogenizer (Bandelin, Germany). After centrifuging at 5000 *g* for 30 min, the supernatant was collected in a 25‐mL container. This process was repeated twice for the precipitate. All the supernatants were then diluted to 25 mL with methanol. Finally, before injection, the supernatants were passed through a 0.45‐μm syringe filter (PTFE or Nylon membrane, suitable for aqueous‐organic solutions).

### 2.5 Soluble Sugars Identification by HPLC

For the quantitative analysis of soluble sugars, a high performance liquid chromatography (HPLC) system (KNAUER/AZURA, Germany) was employed. The system comprised a single piston pump (Smartline 1000; KNAUER, Germany) to deliver the mobile phase, coupled with a refractive index (RI) detector (Smartline RID‐2300) for analyte detection. Separation was achieved using a Eurokat PB column (300 mm × 8 mm; KNAUER, Germany), utilizing an isocratic mobile phase consisting of acetonitrile and deionized water in a 65:35 (v/v) ratio. The mobile phase was delivered at a constant flow rate of 1.8 mL/min. The column temperature was maintained at 65°C to optimize separation efficiency. A sample injection volume of 20 μL was utilized. The total chromatographic run time was 20 min. Analyte identification was based on the principle of RI detection, which measures the difference in RI between the mobile phase and the eluting analytes. Pure sugar standards, including glucose, fructose, and sucrose, were prepared at known concentrations and injected to establish retention time references. Sugars present in the experimental samples were subsequently identified by comparing their retention times to those of the authentic standards. Data acquisition, integration, processing, and storage were performed using EZChrom Elite software (Germany).

### 2.6 Organic Acids Identification by HPLC

Ascorbic acid and citric acid were analyzed using a high performance liquid chromatography (HPLC) system (KNAUER/AZURA, Germany). The HPLC system consisted of a Smartline 1000 single piston pump (KNAUER, Germany), a Smartline 2600 UV detector (KNAUER, Germany), and a Eurospher II 100‐5 C18 column (250 mm × 4.6 mm) for analyte separation. A 50 mM phosphate solution was prepared by dissolving 6.8 g of potassium dihydrogen phosphate (KH_2_PO_4_) in 900 mL of distilled water. The pH of this solution was adjusted to 2.8 with phosphoric acid (H_3_PO_4_), and the solution was then diluted to a final volume of 1000 mL with distilled water, serving as the mobile phase. The analysis was performed under isocratic conditions at a column temperature of 10°C and a flow rate of 0.7 mL/min. For simultaneous detection of the analytes, the UV detector was set to wavelengths of λ = 210 nm for citric acid and λ = 245 nm for ascorbic acid. Prior to sample injection, the separation column was equilibrated with the mobile phase until a stable baseline was achieved. Pure organic acid standards (citric acid and ascorbic acid) were prepared at specific concentrations, and their retention times were recorded. Sample injections were carried out with an injection volume of 20 μL, and the total chromatographic run time for each analysis was 20 min. Organic acids present in the samples were identified based on the matching of their retention times with those of the pure standards. EZChrom software was used for data processing and analysis.

### 2.7 Phenolic Compounds Identification by HPLC

Phenolic compounds, including coumaric acid, *trans*‐ferulic acid, chlorogenic acid, catechin, quercetin, hesperidin, and hesperetin, were identified and quantified using a high performance liquid chromatography (HPLC) system (Agilent Technologies 1200 series, Germany). The system was equipped with a Quaternary pump (Agilent 1260 Infinity, G1311A, USA), and ChemStation software (Agilent Technologies (2001–2009), Rev.B.04.02.96, USA) for data acquisition and processing. Chromatographic separation was achieved using a Zorbax Eclipse C18 column (5 μm, 4.6 × 150 mm) identified using a UV detector (Agilent 1200 Infinity, G1314B, USA). Spectral measurements were conducted at a wavelength of λ = 280 nm, with a mobile phase flow rate of 1 mL/min. The mobile phase gradient consisted of: methanol: 1% formic acid (10:90) for 0 min, methanol: 1% formic acid (25:75) for 10 min, methanol: 1% formic acid (60:40) for 20 min, and methanol: 1% formic acid (70:30) for 30 min. Prior to sample injection, the separation column was equilibrated with the mobile phase until a stable baseline was obtained. Pure phenolic compound standards were injected at defined concentrations, and their respective retention times and absorption spectra were recorded. Sample injections were performed with a volume of 20 μL, and the total chromatographic run time was 40 min. Phenolic compounds present in the samples were identified by comparing their retention times and absorption spectra to those of the authentic standards.

### 2.8 Calibration Curves

Calibration curves were constructed by injecting pure standards at various concentrations. A series of standards with at least five concentration levels was prepared for each compound. The linear range, limit of detection, and limit of quantification were determined for each compound. Subsequently, peak area versus standard concentration was plotted. The performance of the calibration curves was evaluated by calculating the coefficient of determination (*R* ^2^). An *R* ^2^ value close to 1 indicates good linearity of the calibration curve.

### 2.9 Data Analysis

The current study was carried out based on a complete randomized design with five treatments and three replicates. Statistical analysis was performed using a two‐way analysis of variance (ANOVA) procedure in SAS v. 9.4 (Statistical Analysis System, SAS, Cary, NC, USA). This method allows for the simultaneous evaluation of the main effects of each factor, as well as the interaction between them, thereby providing a comprehensive understanding of their influence. The least significant difference (LSD) test was employed following a statistically significant ANOVA result to identify pairwise differences between storage time, postharvest treatment, and the interaction of storage time and postharvest treatment (*p* < 0.05). In addition, R v3.4.3 was used to perform diagrams, Pearson correlation analysis, and principal component analysis (PCA).

## 3 Results and Discussion

### 3.1 Effect of Melatonin and Arginine on Soluble Sugars in Orange Peel

**FIGURE 1:**
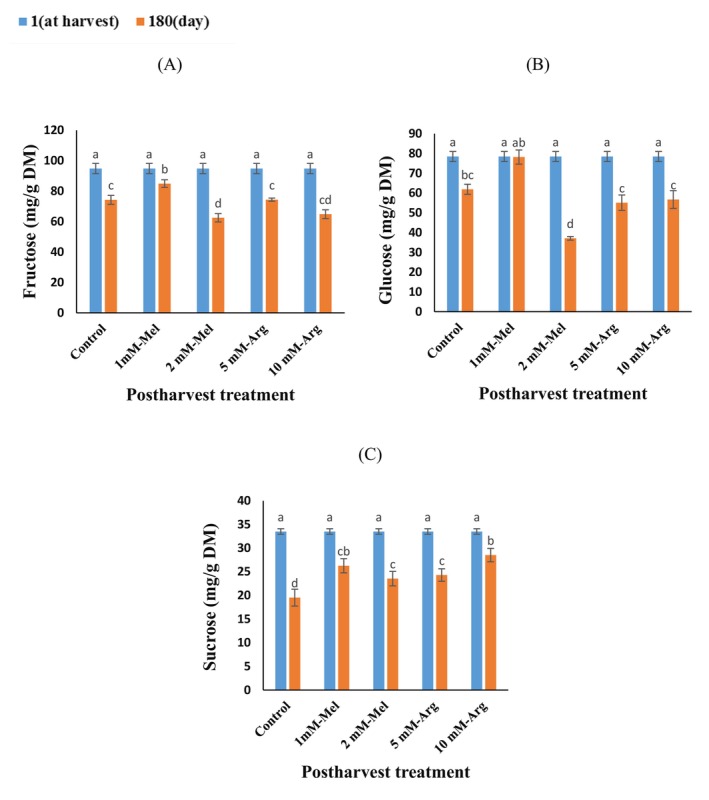
The interaction effect of storage time and postharvest treatment on soluble sugars in “Valencia” orange peel during storage (5°C, 85%–95% RH) for 6 months. Fructose (A), Glucose (B), Sucrose (C). Treatments included melatonin (Mel) and arginine (Arg). Vertical bars on columns represent ± standard error (SE) of means. Data are presented as means. Similar small letters indicate nonsignificant differences at the 5% level of probability using Fisher's least significant difference (LSD) test. The contents of phenolic compounds are expressed on a dry matter basis (mg/g DM).

The study revealed that the levels of sucrose, fructose, and glucose in orange peel were significantly influenced by the postharvest treatments and the interaction of storage time and postharvest treatments. Additionally, storage time significantly affected the content of sucrose and fructose. In this study, the main soluble sugars in orange peel were found to be fructose, followed by glucose. The fructose content decreased in orange peel during cold storage, the treatment with 1 mM‐Mel treatment, which showed a 14% increase compared to the control, whereas the lowest was seen in the 2 mM‐Mel treatment (Figure 1A). However, no significant differences were found between the control, 5 mM‐Arg, and 10 mM‐Arg treatments (Figure 1A). The glucose content fell in the control, 2 mM‐Mel 5 mM‐Arg, and 10 mM‐Arg treatment, and stayed the same in the 1 mM‐Mel treatments at the end of the storage period (Figure 1B). Over the storage period, the sucrose content decreased in all treatments. The postharvest treatments were effectively maintained the sucrose content in orange peel during cold storage (Figure 1C). After 6 months of storage, the lowest sucrose content was observed in the control group, whereas the highest was seen in the 10 mM‐Arg treatment, which showed a 46% increase compared to the control (Figure 1C).

The carbohydrates found in orange peels, such as glucose, fructose, sucrose, pectin, cellulose, and hemicellulose, can be utilized as excellent raw materials for producing biological biofuels like ethanol and biogas (Rivas et al. 2008). The total sugar content in orange peel ranges from 29% to 44%, with glucose, fructose, and sucrose being the primary sugars, along with small amounts of xylose (Rivas et al. 2008). Enzymes such as acid invertase (AI), neutral invertase (NI), sucrose phosphate synthase (SPS), and sucrose synthase (SS) regulate the synthesis and hydrolysis of sucrose into glucose and fructose within distinct cellular compartments. The synthesis of sucrose is mainly managed by SS and SPS, whereas AI, SS cleavage, and NI are involved in the hydrolysis of sucrose into glucose and fructose (Zhang et al. 2019). The 1 mM‐Mel treatment significantly increased fructose and sucrose contents during postharvest storage compared to the control. This elevation in the 1 mM treatment may be attributed to the influence of melatonin on the conversion of organic acids through the gluconeogenesis pathway and other physiological processes, such as cell wall and starch hydrolysis (Echeverria and Valich 1989). Furthermore, Zacarías‐García et al. (2023) reported a 35%–45% increase in glucose content in the pulp extract of Navel, Kirkwood, Valencia, and Ruby oranges during cold storage, which observed a similar trend to the 1 mM‐Mel treatment used in our experiment. The decrease in sucrose content during storage is attributed to its hydrolysis into simple sugars (Echeverria and Valich 1989). Furthermore, the pericarp's ample oxygen supply, which results in the consumption of substrates, promotes cellular respiration because it is in direct contact with the surrounding storage air (Sun et al. 2012). The decline in fructose, glucose and sucrose content in all treatments were likely due to respiration during peel senescence and the consumption of sugars in the respiratory process for production of ATP during storage (Habibi et al. 2020). These findings are in accordance with Sun et al. (2012), who observed a decrease in sucrose concentration in the pericarp of Guanxi pomelo over time. Furthermore, after 10 days of storage, the Satsuma mandarin peel's soluble sugar content decreased. Similar to this trend, a decrease in soluble sugars was also observed in our study. This decrease is likely related to increased respiration during the skin aging process. The decrease in respiration rate in 1 mM‐Mel and 10 mM‐Arg treatments could directly lead to the maintenance of high levels of these sugars (Wu et al. 2019). According to O'Hara et al. (2013), soluble sugar plays an essential role in controlling the growth and development of fruits and plant disease response (Machado et al. 2015). Typically, the pericarp's senescence process is connected to an increase in particular metabolites that are linked to the response to senescence stress (Zhu et al. 2016). In the current study, postharvest treatments led to a lower reduction in sucrose content during cold storage than the control. The higher sucrose content in orange peel treated with melatonin, compared to the control, is likely due to melatonin's ability to prevent the degradation of sucrose into reducing sugars, thus maintaining a higher sucrose level (Chen et al. 2022). Furthermore, the increased sucrose content due to melatonin application may be attributed to the increased activity of SPS and SSs, and the suppression of the sucrose synthase cleavage, AI, and NI activities (Fan et al. 2022). This increase in sucrose levels, relative to the control, aligns with findings reported in previous studies, corroborating the observed elevation in our treatments.

According to the results, the highest sucrose content was found in orange peel treated with 10 mM‐Arg, suggesting that a higher concentration of arginine can more effectively promote sucrose content during cold storage compared to other treatments. The increased sucrose content in the peel treated with arginine, compared to the control, may be due to a slower reduction of soluble sugars during cold storage; this observation is consistent with our findings (Li, Ding, et al. 2019). The glucose content in the peel of oranges treated with control, 1 mM‐Mel treatments did not exhibit significant changes over 6 months of storage, perhaps as a result of comparable consumption and synthesis rates (Cukrov et al. 2016). However, it was observed that the 1 mM‐Mel concentration resulted in the highest levels of glucose compared to other treatments, 2 mM‐Mel, 5 mM‐Arg, and 10 mM‐Arg, in the orange peel. This outcome could be attributed to melatonin's regulation of the levels of transcript for the SPS, SS, NI, and AI enzymes as well as their activities (Zhang et al. 2023). It has been reported that melatonin stimulates the activities of enzymes related to sucrose metabolism, leading to higher contents of glucose and fructose. Our findings are consistent with Liu et al. (2020) and Miranda et al. (2020), who reported that postharvest changes in soluble sugar content of mango and sweet cherry were effectively suppressed by exogenous melatonin; this observation is consistent with our study at a 2 mM‐Mel concentration. Previous research has indicated that exogenous arginine significantly slowed the decline in total sugar levels in white button mushrooms (*Agaricus bisporus*) and soluble sugars in sweet pepper fruits compared with the control; this observation deviated from the findings of our study (Akram et al. 2021; Li, Ding, et al. 2019).

### 3.2 Effect of Melatonin and Arginine on Organic Acids in Orange Peel

**FIGURE 2:**
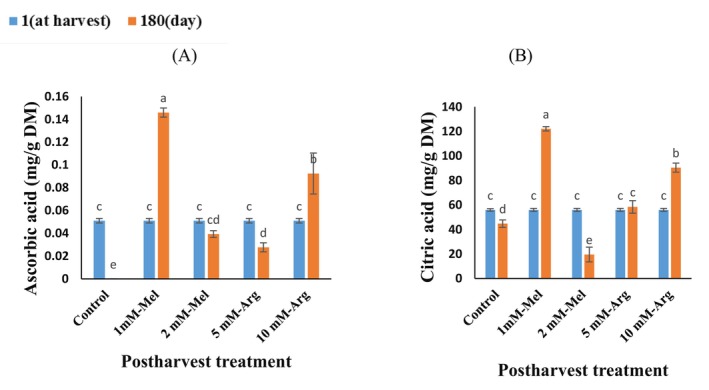
The interaction effect of storage time and postharvest treatment on organic acids content in “Valencia” orange peel during storage (5°C, 85%–95% RH) for 6 months. (A) Ascorbic acid, (B) Citric acid. Treatments include melatonin (Mel), and arginine (Arg). Vertical bars on columns represent ± standard error (SE) of means. Data are represented as means. The similar small letters indicate nonsignificant differences at the 5% level of probability according to Fisher's least significant difference (LSD) test. The contents of phenolic compounds are expressed on a dry matter basis (mg/g DM).

The storage time, postharvest treatment, and their interaction significantly influenced the citric acid and ascorbic acid content in orange peel. The ascorbic acid content rose in the 1 mM‐Mel and 10 mM‐Arg treatments at the end of storage, but it fell in the control, 2 mM‐Mel, and 5 mM‐Arg treatments (Figure 2A). The highest ascorbic acid content was found in the 1 mM‐Mel (Figure 2A). Throughout the storage period, citric acid content increased in 1 mM‐Mel and 10 mM‐Arg treatments at the end of storage, but decreased in the control and 2 mM‐Mel treatments and remained constant in the 5 mM‐Arg treatment (Figure 2B). After 6 months of storage, the 1 mM‐Mel and 10 mM‐Arg treatments resulted in a 173% and 102% increase in citric acid content compared to the control, respectively (Figure 2B).

Orange peel contains approximately 1% organic acids, including ascorbic acid and citric acid. Citric acid is a key industrial chemical extensively used in the food and beverage, pharmaceutical, and cosmetic industries. With the increasing demand for sustainable and eco‐friendly production methods, there is growing interest in utilizing waste materials as feedstocks for citric acid production (Teigiserova et al. 2022). Orange peel waste, a by‐product of the orange juice industry, is one such material. Producing citric acid from orange peel waste offers a promising approach to sustainable and eco‐friendly production. With further research and development, this process could become a viable alternative to traditional citric acid production methods (Mohsin et al. 2022). Thus, preserving high levels of organic acids or slowing their degradation in orange peel could be crucial.

Throughout postharvest storage, the outer layer of the fruit, known as the pericarp, consumes substrates through respiration to generate energy to withstand resistance to microorganisms and various stresses. Unfortunately, this leads to a loss of nutrients and a decline in quality, which is not ideal for consumers. Both during cold storage and after harvest, citrus fruits break down organic acids and sugars to sustain cellular metabolism. The primary energy source's consumption is probably the cause of the decrease in organic acids during cold storage (Daneshvar et al. 2023). During respiration, organic acids are converted into sugars, water, carbon dioxide, calcium and potassium salts, and other compounds (Wu et al. 2014). Additionally, the reduction in organic acids may be linked to fruit senescence after long‐term storage (Habibi et al. 2020). In our investigation, the citric acid content decreased in the control, 2 mM‐Mel, and remained constant in the 5 mM‐Arg treatment. During the cold storage period, ascorbic acid content also decreased in the control, 2 mM‐Mel, and 5 mM‐Arg treatments. Ascorbic acid is highly susceptible to chemical and enzymatic oxidation during the storage of horticultural crops (Hassan and Hussein 2017). The degradation of ascorbic acid over time can be attributed to direct oxidative breakdown due to ascorbinase activity or indirect degradation through the activities of cytochrome oxidase, PPO, and peroxidase (Hassan and Hussein 2017).

According to reports, L‐ascorbic acid converts into dehydro‐ascorbic acid, which can lead to a decrease in ascorbic acid content during cold storage (Choudhary et al. 2008). This reduction in ascorbic acid content could be associated with the overproduction of reactive oxygen species (ROS), as ascorbic acid serves as an electron donor to neutralize ROS during storage (Habibi et al. 2020). Johnston and Bowling (2002), research has indicated a notable reduction in the ascorbic acid levels in orange juices, even in unopened containers. Additionally, Sun et al. (2012) reported declines in malic acid, citric acid, and quinic acid concentrations in the pericarp (albedo and flavedo) of Shatian pumelo ( *Citrus grandis* ) and Hirado Butun pumelo (*C. grandis × Citrus paradisi*). Similarly, Gao et al. (2018) found reduced citric acid content in navel oranges during storage. Overall, studies indicate that the total organic acid content in citrus fruits progressively decreases as storage time extends (Chen et al. 2022) Our results align with those reported in previous studies. Exogenous melatonin application has been shown to delay postharvest senescence and maintain higher levels of organic acids. For instance, melatonin treatment in pomegranate trees resulted in higher total acidity and better quality during storage compared to untreated fruits (Medina‐Santamarina et al. 2021) These findings are consistent with our observations. Melatonin may regulate metabolic pathways that influence organic acid levels, potentially slowing down the degradation processes associated with fruit senescence (Zhao et al. 2024) These studies corroborate our findings. In the current study, it was found that treatment with 1 mM‐Mel and 10 mM‐Arg effectively increased ascorbic acid content, whereas treatment with 2 mM‐Mel was effective in maintaining the ascorbic acid compared to the control during the storage. In addition, the reduction of citric acid content was lower in the 2 mM‐Mel treatments compared to the control. The accumulation of organic acids is linked to the tricarboxylic acid (TCA) cycle (Zhang et al. 2019). It is speculated that the TCA cycle might be affected by the low concentration of melatonin and the high concentration of arginine, leading to increased ascorbic acid content in the 1 mM‐Mel and 10 mM‐Arg treatments during cold storage. The observed effect of the 2 mM‐Mel treatments in maintaining ascorbic acid content as well as the 1 mM‐Mel and 10 mM‐Arg treatments in preserving citric acid in orange peel could be attributed to a reduction in metabolic activity. This may explain the smaller reduction in citric and ascorbic acid content compared to the control during cold storage (Wang et al. 2017). The application of melatonin can effectively delay physiological senescence by reducing postharvest respiration and functioning as an excellent antioxidant in plants (Onik et al. 2021). Furthermore, melatonin inhibits ascorbic acid oxidase (AAO) activity, leading to higher levels of ascorbic acid in treated fruits (Gao et al. 2016; Liu et al. 2016).

According to reports, fruits treated with arginine may have higher ascorbic acid contents because of decreased oxidation and ascorbinase activity (Khan et al. 2023). The glutathione reductase (GR)/ascorbate peroxidase system's increased activity or the AAO enzyme's decreased activity have also been proposed as the causes of the ascorbic acid content increase (Babalar et al. 2018). These findings align with the studies by Bal (2019) on plum, Ba et al. (2022) on pitaya, and Shekari et al. (2021) on mushrooms (*Agaricus bisporus*), all of which demonstrated that the application of exogenous melatonin caused an increase in ascorbic acid in treated crops. Moreover, arginine treatment has been shown to increase the accumulation of ascorbic acid in various fruits, such as cucumber Hasan et al. (2020), guava (Ali et al. 2022), and pomegranate (Babalar et al. 2018).

### 3.3 Effect of Melatonin and Arginine on Phenolic Compounds in Orange Peel

**FIGURE 3:**
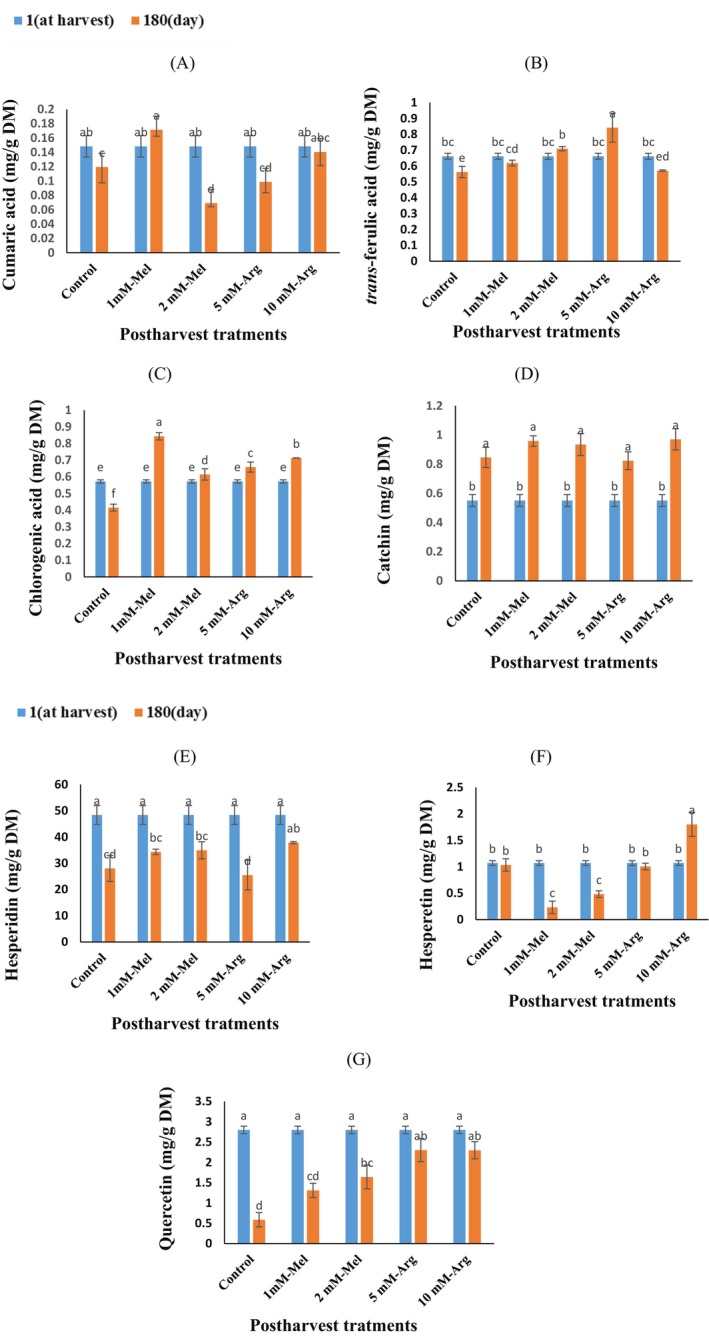
The interaction effect of storage time and postharvest treatment on phenolic compounds in ʿValenciaʾ orange peel during the storage (5°C, 85%–95% RH) for 6 months. Cumaric acid (A), *trans*‐ferulic acid (B), Cholorogenic acid (C), Catechin (D), Hesperidin (E), Hesperetin (F), Quercetin (G). Treatments include melatonin (Mel) and arginine (Arg). Vertical bars on columns represent ± standard error (SE) of means data are presented as means. The similar small letters indicate nonsignificant differences at the 5% level of probability using Fisher's least significant difference (LSD) test. The contents of phenolic compounds are expressed on a dry matter basis (mg/g DM).

The results revealed that storage time, postharvest treatment, and their interaction had a significant impact on the phenolic compounds in orange peel during cold storage. Specifically, the levels of coumaric acid, *trans*‐ferulic acid, chlorogenic acid, catechin, quercetin, hesperidin, and hesperetin were affected. Hesperidin was identified as the primary phenolic compound, followed by quercetin (Figure 3). As shown in Figure 1A, the coumaric acid content in orange peel decreased in the control, 2 mM‐Mel, and 5 mM‐Arg treated fruits during storage, whereas it remained unchanged in the 1 mM‐Mel and 10 mM‐Arg treated ones. The most effective treatments in retaining coumaric acid were the 10 mM‐Arg and 1 mM‐Mel treatments (Figure 3A). Following a 6‐month storage period, the *trans*‐ferulic acid content increased in 5 mM‐Arg treatment, stayed constant in the 1‐mM‐Mel and 2 mM‐Mel treatments, and decreased in the control and 10‐mM‐Arg treatments (Figure 3B). The 5 mM‐Arg treatment showed the highest increase, with a 50% rise compared to the control (Figure 3B). As shown in Figure 3C, the chlorogenic acid content increased with postharvest treatments but decreased in the control. The highest chlorogenic acid content was associated with the 1‐mM‐Mel treatment, which showed a 103% increase compared to the control.

After 6 months of storage, catechin content increased in all treatments, with the highest content observed in the 10 mM‐Arg treatment, exhibiting an 15% increase compared to the control. No significant difference was found between the other treatments (Figure 3D). Orange peel's hesperidin content decreased during cold storage, However, the 10 mM‐Arg treatment showed a 35% increase compared to the control (Figure 3E). After 6 months of storage, the hesperetin content increased in the 10 mM‐Arg treatment, remained unchanged constant in the control and 5 mM‐Arg treatments, and decreased in the 1 mM‐Mel and 2 mM‐Mel treatments (Figure 3F). The 10 mM‐Arg treatment resulted in a 74% increase in hesperetin content compared to the control, as shown in Figure 3F. The quercetin content of orange peel decreased in the control, 5 mM‐Arg, 10 mM‐Arg and 2 mM‐Mel treatments at the end of storage, but the 10 mM‐ Arg treatment showed no significant change (Figure 3G). The highest effectiveness in maintaining quercetin content was attributed to the 10 mM‐ Arg treatment (Figure 3G).

Orange peels contain a high concentration of bioactive compounds, including flavonoids and other phenolic compounds. These compounds have potential applications in producing biochemicals such as antioxidants, antimicrobials, and nutraceuticals. Flavonoids can be categorized into flavones (apigenin, luteolin, rutin), flavanones (hesperidin, naringin, naringenin), flavonols (quercetin, kaempferol), isoflavones, and catechins (Gattuso et al. 2007; Sharma et al. 2019). The primary flavonoids in the peel of orange are hesperidin, naringin, neohesperidin, narirutin, rutin, and quercetin (Sawalha et al. 2009), with hesperidin being the most abundant (Sharma et al. 2019). Hesperidin, in particular, offers numerous therapeutic benefits due to its antioxidative, anti‐inflammatory, and anticarcinogenic properties (Espinosa‐Pardo et al. 2017). Additionally, numerous phenolic acids, including caffeic acid, chlorogenic acid, *trans*‐ferulic acid, cinnamic acid, and p‐coumaric acid are commonly found in orange peels (Petrotos et al. 2021). The extraction and production of bioactive compounds from orange peels have gained traction due to the increased global demand for herbal products. Consequently, orange peel is considered a sustainable and cost‐effective source for producing value‐added biochemicals.

The reduction in total phenols content during storage might be attributed to the oxidative activity of the enzyme PPO, which converts phenols into quinones (Babalar et al. 2023). At the end of storage, the control group's phenolic compound levels may have decreased due to a decrease in phenolic content during fruit senescence. In this study, the amount of hesperidin, the primary phenolic compound, significantly decreased in orange peel during storage. This decline in hesperidin could be related to the cessation of the activities of enzymes involved in its biosynthesis, such as chalcone synthase, chalcone isomerase, phenylalanine ammonia‐lyase (PAL), manonyl transferase, and UDP‐rhamnose flavanone glucoside rhamnosyltransferase (Chao et al. 2022). Furthermore, this trend might be due to the lack of essential precursors for flavonoid production, like malonyl‐coenzyme and phenylalanine (Barreca et al. 2017). These findings align with the results of previous studies on “Valencia” orange peel by (Shamloo et al. 2015) and on the pericarp of *Citrus reticulata* “Chachi” (at immature stage) by Chao et al. (2022), which reported a decrease in hesperidin content during prolonged storage. Similarly, Nam et al. (2019) report that during storage, the hesperidin content of mandarin oranges (*Citrus unshiu* (Swingle) Marcov) also decreased. Gene expression in the phenylpropanoid pathway, including PAL, has been shown to be regulated by melatonin (Wang et al. 2020) and has been utilized to enhance the levels of beneficial compounds such as sucrose, natural antioxidants, and phenolic compounds, aroma components, polyphenolics, and soluble solids in fruits (Xia et al. 2020). One possible explanation for this rise in phenolic compounds is that cold storage raises the phenolic content by activating phenolic‐associated genes (Zhang et al. 2016). Melatonin has been reported to regulate gene expression in the pathway of phenylpropanoid, such as PAL. This stimulation of the phenylpropanoid pathway leads to the accumulation of total phenolics in postharvest fruits (Zhang et al. 2016). Additionally, melatonin treatment has been found to increase the activity of enzymes involved in phenolic compound synthesis, such as shikimate dehydrogenase, PAL, and glucose‐6‐phosphate dehydrogenase during postharvest storage (Gao et al. 2018). Exogenous arginine application may suppress senescence during storage, as evidenced by higher levels of phenolic compounds observed compared to control. It has been observed that treating with L‐arginine lowers free radicals and protects the cell wall and membrane by promoting the synthesis of antioxidants, which stops phenolic compounds from being lost postharvest (Shu et al. 2020). Furthermore, the generation of internal nitric oxide and the breakdown of free radicals by L‐arginine treatment increases the amount of phenolic compounds, which in turn improves stress resistance (Li, Ding, et al. 2019). Previous research has shown that plants use the shikimate pathway for the synthesis of ferulic acid. This process either starts with tyrosine directly converting into p‐coumaric acid, or it starts with phenylalanine being converted into cinnamic acids and then into p‐coumaric acid (Wu et al. 2020). Eventually, p‐coumaric acid transforms into ferulic acid through hydroxylation and methylation (Kumar and Pruthi 2014). The exogenous application of melatonin and arginine may have upregulated the shikimate pathway, which could account for the increase in *trans*‐ferulic acid observed in the 2 mM‐Mel and 5 mM‐Arg treatments during storage.

Research has indicated that treating fruit with melatonin leads to an increase in epicatechin, catechin, coumaric acid, chlorogenic acid, and ferulic acid levels within grape berries, thereby boosting the overall phenolic content of the fruit (Xu et al. 2017) Our study is in agreement with these investigations. High concentrations of arginine (10 mM) during storage have also been shown to increase phenolic compounds, including gallic acid, catechin, quercetin, *trans*‐ferulic acid, and chlorogenic acid, due to the upregulation of PAL activity and downregulation of PPO enzyme activity (Babalar et al. 2018). Researchers have found that pomegranate fruit showed reduced chilling injury in response to arginine treatment, leading to an increase in phenolic compounds because of increased PAL and decreased PPO enzyme activity (Babalar et al. 2018). Similarly, Li et al. (2019) demonstrated that arginine increased the total phenolic content in white mushrooms during storage, possibly through the inhibition of PPO activity. Furthermore, arginine treatment was found to improve the total phenol content in strawberries and tomatoes this observation is in agreement with our current study (Shu et al. 2020).

### 3.4 PCA and Pearson Correlation Analysis

**FIGURE 4:**
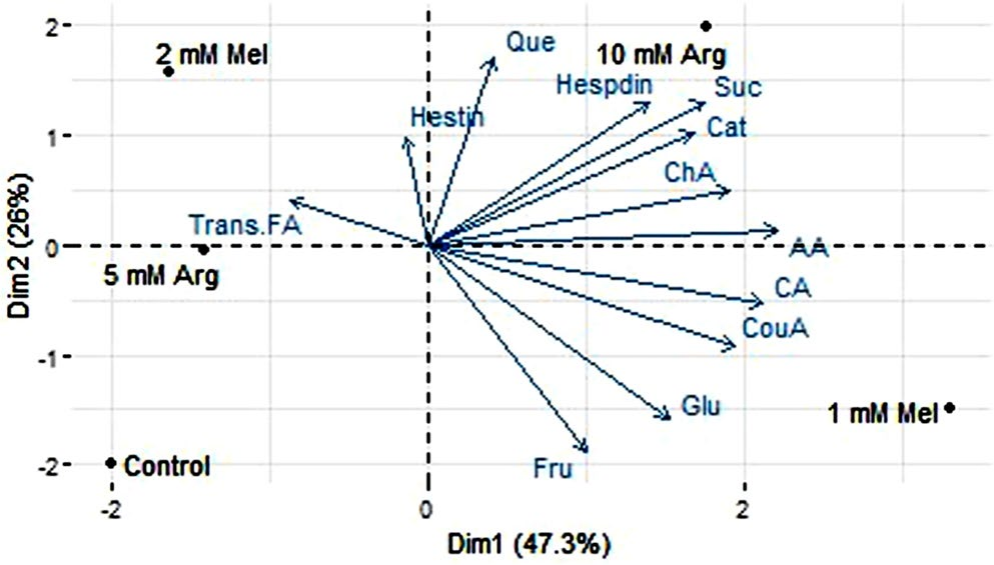
Principal component analysis (PCA) of database obtained from postharvest melatonin and arginine treatments in “Valencia” orange fruit after 6 months storage. (Dim1 and Dim2): Axes represent the principal directions of variation within the data. Variable Vectors: Vector in the biplot represents a measured variable. The length of the vector indicates the magnitude of its contribution to the principal components. The direction of the vector reflects the correlation of the variable with the principal components. Treatment Points: Points in the biplot represent individual experimental treatments. AA, ascorbic acid; CA, citric Hesdin, hesperidin; Cat, catechin; ChA, chlorogenic acid; CouA, coumaric acid; Fru, fructose; Glu, glucose; Hestin, hesperetin; Que., quercetin; Suc, sucrose; Trans.FA, *trans*‐ferulic acid.

**FIGURE 5:**
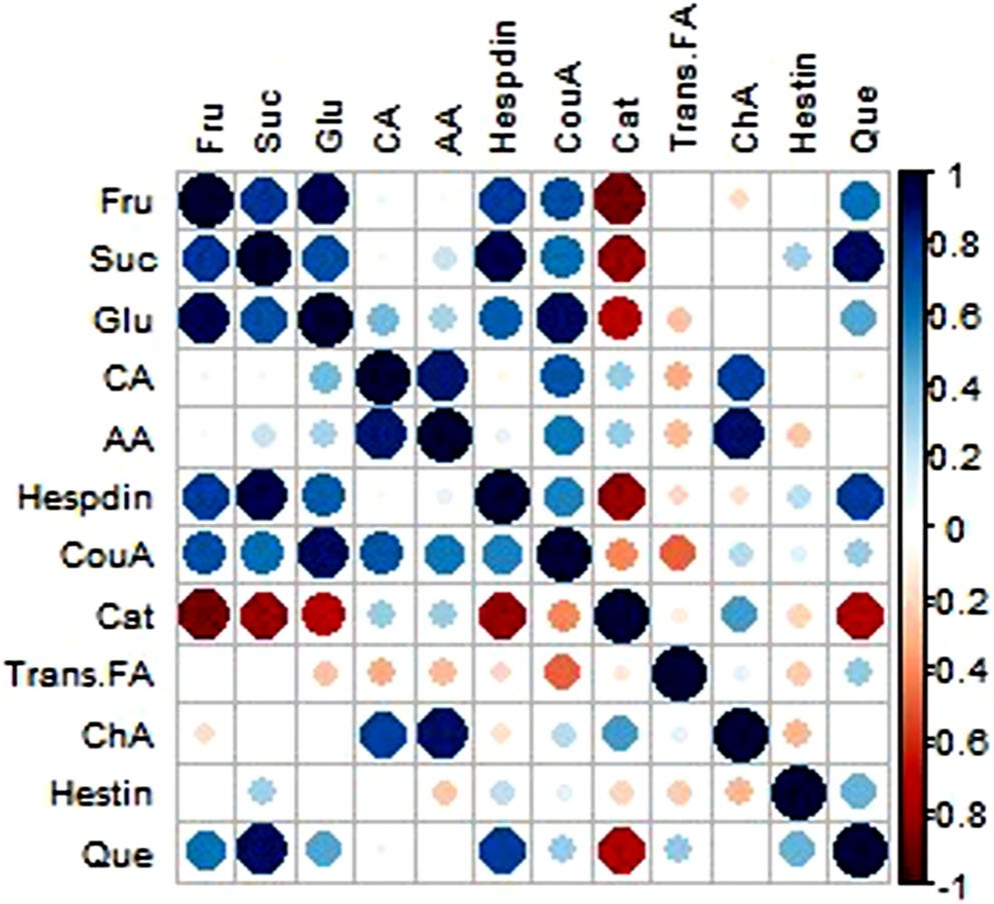
Depicting correlation coefficients (*r*) ranging from −1.0 to +1.0. Positive and negative values are represented in red and blue, respectively. AA, ascorbic acid; CA, citric acid; Cat, catechin; ChA, chlorogenic acid; CouA, coumaric acid; Dim1, dimension1; Dim2, dimension2; Fru, fructose; Glu, glucose; Hesdin, hesperidin; Hestin, hesperetin; Que, quercetin; Suc, sucrose; Trans.FA, *trans*‐ferulic acid.

The PCA analysis revealed associations among variables after 6 months of storage (Figure 4). PCA showed the relationship between variables after applying treatments (Figure 4). The principal axes include the horizontal axis (Dim1) which explains 47.3% of the variance of the data, and the vertical axis (Dim2) which covers 26% of the variance. In (Figure 4), the factors quercetin, hesperidin, sucrose, catechin, and chlorogenic acid have high correlation with the Dim1 axis and are located on the right side. On the other hand, variables such as glucose and fructose show high correlation with the negative part of Dim1. Other factors such as citric acid, coumaric acid, and ascorbic acid are also located on the positive side of Dim1, indicating that the increase of these variables is associated with certain treatments such as 10 mM Arg. In contrast, transferulic acid has a higher correlation with Dim2. The Pearson correlation heatmap showed the positive correlations among citric acid content with ascorbic acid, as well as glucose with sucrose (Figure 5). Additionally, Figure 4B shows that *trans*‐ferulic acid had a positive correlation with chlorogenic acid and catechin. Furthermore, the content of catechin had a negative correlation with hesperidin content in “Valencia” orange peel after 6 months of storage (Figure 5).

## 4 Conclusion

**FIGURE 6:**
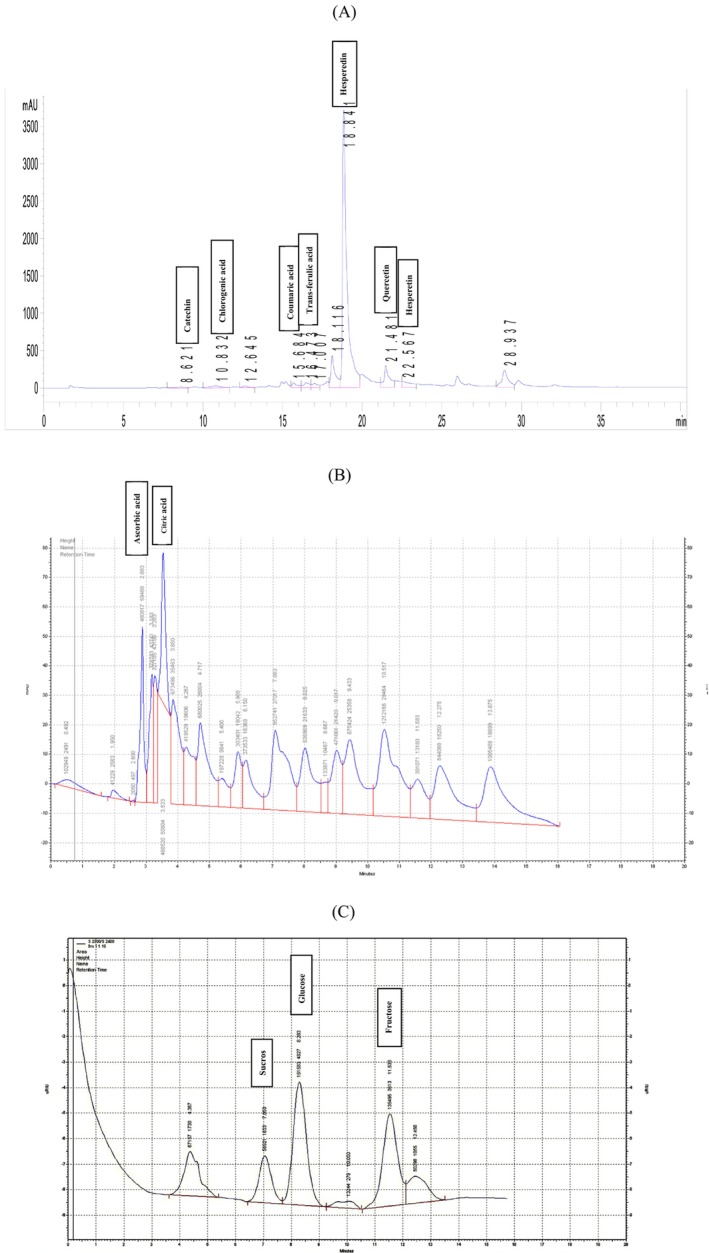
HPLC chromatogram in ʿValenciaʾ orange peel during the storage (5°C, 85%–95% RH) for 6 months: phenolic compounds (A), organic acids (B), sugars (C).

The present study demonstrated that postharvest application of melatonin and arginine not only acts as preservatives but also as active stimulants to enhance and stabilize the biochemical characteristics of Valencia orange peel. After 6 months of storage, a number of control fruits developed fungal rot. During storage, the degree of fruit rot was monitored regularly and the last measurement was made when approximately 50% of the fruits were contaminated and rotted. This contamination was observed first in control fruits and then in the 5 mM‐Arg treatment. These treatments selectively and concentration‐dependently improved the nutritional and functional quality of orange peel under cold storage conditions. The chromatographic profiles clearly reflect organic acids, soluble sugars, and phenolic compounds shift influenced by postharvest treatments (Figure 6). Notably, the different effects of different concentrations of arginine and melatonin on the increase and maintenance of specific compounds highlight the precise regulatory role of these substances in the physiological responses of the plant to postharvest conditions. In particular, 1 mM‐Mel and 10 mM‐Arg effectively increased and maintained the levels of organic acids, soluble sugars, and coumaric acid, chlorogenic acid contents, and also had a significant effect on the proper appearance of the fruit compared to the control fruits. Also, 1 mM‐Mel, 2 mM‐Mel, and 5 mM‐Arg effectively increased and maintained the levels of trans‐ferulic acid, whereas 10 mM‐Arg maintained the levels of hesperidin. Therefore, the use of these postharvest treatments could provide a promising approach to maintain the quality of orange peel waste and increase the production of high value‐added products for pharmaceuticals, nutraceuticals, health drinks, cosmetics, and various other fields. These findings, beyond confirming the importance of melatonin and arginine in maintaining fruit quality, illuminate their potential as tools for designing innovative strategies to increase the added value of agricultural products and reduce postharvest losses.

## Author Contributions

**Faezeh Aghaei:** formal analysis (equal), funding acquisition (equal), methodology (equal), software (equal), supervision (equal), writing – original draft (equal). **Asghar Ramezanian:** conceptualization (equal), funding acquisition (equal), investigation (equal), methodology (equal), project administration (equal), resources (equal), supervision (equal), validation (equal), writing – review and editing (equal). **Mohammad Jamal Saharkhiz:** conceptualization (equal), funding acquisition (equal), investigation (equal), methodology (equal), project administration (equal), resources (equal), supervision (equal), validation (equal), writing – review and editing (equal). **Mohammad‐Taghi Golmakani:** conceptualization (equal), investigation (equal), methodology (equal), supervision (equal), validation (equal), writing – review and editing (equal). **Vahid Rowshan:** Conceptualization, investigation (equal), methodology (equal), supervision (equal), validation (equal), writing ‐ review and editing (equal).

## Conflicts of Interest

The authors declare no conflicts of interest.

## Data Availability

The data that support the findings of this study are available on request.
